# Supplementary material for: DNA Methylation Profiles of PSMA6, PSMB5, KEAP1, and HIF1A Genes in Patients with Type 1 Diabetes and Diabetic Retinopathy
Source: Biomedicines. 2024 Jun 18;12(6):1354. doi: 10.3390/biomedicines12061354 (PMC11202151; doi:10.3390/biomedicines12061354)
Supplement: Supplementary file 1 [file biomedicines-12-01354-s001.zip › biomedicines-3015297-supplementary.pdf]

biomedicines

MDPI

Table S1 Primers and methodological details of the *PSMA6*, *PSMB5*, *HIF1A*, and *KEAP1* region-specific DNA methylation study.

Gene Symbol/ID	Description of the Gene:	LEFT PRIMER 5'-3'	CpG Island Position:	Product size, bp	Rest. Sites, *n*
PSMA6/5687	proteasome 20S subunit alpha 6	F_CATGCAAGAGCGGAAGAAAC<br>R_GGTAATGTGGCGGTCAAAAC	chr14:35292195-35292648	171	4
PSMB5/5693	proteasome subunit beta type-5 isoform 1	F_ATCTGGCTCTTCTTTTTGGA<br>R_TCCATGAAGCATTTCGATT	chr14:23034690 - 23035039	332	5
HIF1A/3091	hypoxia inducible factor 1 alpha subunit	F_CACCCCCATCTCCTTTCTCT<br>R_GGGTTCCTCGAGATCCAATG	chr14:61695130-61696513	171	2
KEAP1/9817	kelch like ECH associated protein 1	F_GAAACCGAGCGAGAGAACC<br>R_ACTTCCCCCACCCTACAAAG	chr19:10502371-10503604	199	2

n-number of restriction sites in the amplified regions of promoter parts of investigated genes, for the enzymes present in the OneStep qMethyl kit (Zymo Research, www.zymoresearch.com); CpG Islands positions are indicated according to localization on the chromosome.
